# Flurochloridone Induced Cell Apoptosis via ER Stress and eIF2α-ATF4/ATF6-CHOP-Bim/Bax Signaling Pathways in Mouse TM4 Sertoli Cells

**DOI:** 10.3390/ijerph19084564

**Published:** 2022-04-11

**Authors:** Fen Zhang, Zhijing Ni, Shuqi Zhao, Yanna Wang, Xiuli Chang, Zhijun Zhou

**Affiliations:** School of Public Health, MOE Key Laboratory for Public Health Safety/NHC Key Lab of Health Technology Assessment, Fudan University, Shanghai 200032, China; 19211020032@fudan.edu.cn (F.Z.); 18211020078@fudan.edu.cn (Z.N.); 20211020178@fudan.edu.cn (S.Z.); 20211020056@fudan.edu.cn (Y.W.); xlchang@fudan.edu.cn (X.C.)

**Keywords:** Flurochloridone, testicular toxicity, TM4 Sertoli cells, endoplasmic reticulum stress, unfolded protein response, apoptosis

## Abstract

Flurochloridone (FLC), as a novel herbicide, has been widely used in many countries since 1980s. Current studies have shown that FLC has toxic effects on male reproduction and its target organ is testis, while the underlying mechanism is still unknown. Mouse testis Sertoli cell line TM4 cells were used as an in vitro model and treated with FLC at different doses (40, 80, 160 μM) for different times (6, 12, 24 h). Cell viability, cytotoxicity and apoptotic cells were detected by CCK-8 assay, LDH leakage assay and flow cytometry. The protein levels of GRP78, phosphorylated-eIF2α, ATF4, ATF6, CHOP, Bim and Bax were observed by Western Blot and Immunofluorescence staining. FLC inhibited cell viability and induced cytotoxicity in dose-dependent way in TM4 cells. The percentage of apoptotic cells were 6.2% ± 0.6%, 7.3% ± 0.3%, 9.8% ± 0.4%, 13.2% ± 0.2%, respectively. The expression levels of ER stress and UPR related proteins were activated over dose. Meanwhile, the pro-apoptotic proteins (Bim and Bax) were also up-regulated in dose-dependent. After pretreated with ISRIB, the inhibitor of eIF2α phosphorylation, the elevated expression of GRP78, phosphorylated-eIF2α, ATF4, ATF6, CHOP and Bim was down to normal level accordingly. In conclusion, FLC induced apoptosis in TM4 cells mediated by UPR signaling pathways.

## 1. Introduction

As a pyrrolidone herbicide, Flurochloridone (FLC) has been widely used in the European Union and North American countries since 1980s. It is relatively safe for crops and has a huge potential market, mainly in winter wheat, cotton, sunflower and carrot fields to control most broad-leaved weeds [1]. However, more and more concerning on human health risk of FLC is coming with the release of toxicological studies on genotoxicity and reproductive, etc. The target organs of FLC are testis and epididymis in male animals [2]. FLC and its formulations could induce DNA damage [3,4], cytotoxicity [5] and reproductive toxicity [6,7] in vivo and vitro. It was clear that FLC induced the vacuolation of Sertoli cells [6,7]. Sertoli cells are crucially important cells in testis. They can maintain the normal immune environment by constructing blood-testis barrier (BTB) [8] and regulate the process of spermatogenesis [9,10]. Many chemicals can destroy Sertoli cells and even induce cell death [11,12,13]. In addition, many of those chemicals, such as ZnSO_4_, Zearalenone, can cause ER stress in Sertoli cells [14,15]. Studies in vitro showed that FLC induced the accumulation of reactive oxygen species (ROS), disturbed calcium homeostasis, activated ERK1/2 signaling pathway in rat primary Sertoli cells [16], and induced cell apoptosis via mitochondria pathway [17] and autophagy via AKT-mTOR signaling pathway in mouse TM4 Sertoli cells [18]. The evidence so far has suggested that FLC maybe activate endoplasmic reticulum (ER) stress in the process of inducing cell apoptosis.

Under the stimulation of exogenous chemicals, cells will initiate the stress response to resist the toxic effects and maintain the cellular environmental homeostasis. When the exogenous chemicals continue to stimulate cells, cells will undergo apoptosis or death [19]. Endoplasmic reticulum (ER) is a locus where proteins are modified, folded and calcium saved. When misfolded proteins accumulate in ER, ER stress will occur and initiate unfolded protein response (UPR) to clear the misfolded proteins [20,21].There are three UPR signaling proteins attached to the membrane of ER, namely eukaryotic initiation factor 2α (eIF2α) kinase (PERK), activating transcription factor 6 (ATF6) and type I transmembrane protein inositol requiring 1 (IER1α) [22]. Once ER stress occurs, the three UPR signaling proteins separate from immunoglobulin protein/Glucose-Regulated Protein 78 (BIP/GRP78) and activate downstream proteins in different ways. One of the UPR signaling protein, PERK, is activated by autophosphorylation and in turn phosphorylates eIF2α. Phosphor-eIF2α activates the expression of ATF4 to promote the transcription of apoptosis related genes to initiate apoptosis, such as the proapoptotic factor CCAAT/enhancer-binding protein homologous protein (CHOP). ATF6 also can activate the transcription of CHOP [23,24,25]. Long-term activation of CHOP can trigger cell apoptosis and induce cell apoptosis by down-regulating the anti-apoptotic protein B-cell lymphoma-2 (Bcl-2) and up-regulating the pro-apoptotic proteins Bcl-2 interacting mediator (Bim), Bcl-2-associated X protein (Bax) and Bcl-2 homologous antagonist/killer (Bak) [26,27].

The specific mechanism of FLC inducing the injury of Sertoli cells has not been clarified yet. It is necessary to explore the role of ER stress played in FLC-induced apoptosis of Sertoli cells, which can provide scientific evidence for the study of the toxic mechanism of FLC, and also provide treatment methods for alleviating the toxic effects of FLC. The TM4 cell line has been cultured as a proper model for mouse Sertoli cells in vitro and used in scientific research since they possess many similarities to primary Sertoli cells on characterization and gene expression [28,29]. In this study, the role of endoplasmic reticulum stress and the possible UPR signaling pathways (eIF2α-ATF4-CHOP) in FLC-induced apoptosis was explored in TM4 cells.

## 2. Materials and Methods

### 2.1. Cell Culture and Treatments

The TM4 cell line was obtained from National Infrastructure of Cell Line Resource (NICR, Beijing, China) and cultured in Dulbecco’s modified eagle medium (DMEM, Gibco, Waltham, MA, USA) with 10% (*v*/*v*) fetal bovine serum (FBS, Gibco, Waltham, MA, USA) as described previously [17]. FLC was purchased from Duma Biotechnology Co. Ltd. (Shanghai, China) and dissolved with dimethyl sulphoxide (DMSO, Sigma-Aldrich, St. Louis, MO, USA) as described previously [18]. For cell viability assay and cytotoxicity assay, cells were seeded in 96-well plates at a density of 5 × 10^3^ cells/well and there were 6 duplicate wells in each group. For western blot and flow cytometry, cells were seeded in 6-well plates at a density of 2 × 10^5^ cells/well, and there were 3 duplicate wells in each group. Cells were treated with FLC at the concentration of 0, 40, 80, 160 μM for 6, 12, 24 h before experiments. For fluorescence staining of proteins, cells were seeded in 24-well plates with a cell slide in each well. For intervention experiment, cells were pretreated with ISRIB (an inhibitor of eIF2α phosphorylation, purchased from Beyotime Biotechnology, Jiangsu, China, at the concentrations of 200, 400 nM for 6 h) and followed by the treatment of FLC (at the concentration of 160 μM for 6 h). All experiments were repeated at least three times.

### 2.2. Cell Viability Assay

Cell viability assay was tested by cell counting kit-8 (CCK-8 kit, Beyotime Biotechnology, Jiangsu, China) according to the manufacturer’s protocol. Briefly, after TM4 cells treated with FLC or/and ISRIB, the cells in each well were added with 10 μL reagent of CCK-8 kit at scheduled time point and then incubated at 37 °C for 1.5 h protected from light. The optical density (OD) values were measured at the wavelength of 450 nm by a Synergy HT Microplate Reader (BioTek, Winooski, VT, USA). Cell viability was calculated by the relative OD values to the control group.

### 2.3. Cytotoxicity Assay

Cytotoxicity assay was determined by LDH (lactate dehydrogenase) Cytotoxicity Assay Kit (Beyotime Biotechnology, Jiangsu, China) according to the manufacturer’s protocol. In brief, 100 μL cell supernatants from each well were collected and transferred to a new 96-well plate. 50 μL LDH reagent was added to each well and then incubated at 37 °C for 30 min protected from light. The OD values were measured at 490 nm by a Synergy HT Microplate Reader. The cytotoxicity was calculated by the relative OD values to the control group.

### 2.4. Determination of Apoptotic Cells by Flow Cytometry

Apoptotic cells were determined by flow cytometry using Annexin V-FITC Apoptosis Detection Kit (Beyotime Biotechnology, Jiangsu, China) according to the manufacturer’s protocol. After treated with FLC/ISRIB for 6 h, TM4 cells were collected by Trypsin-EDTA solution (Beyotime Biotechnology, Jiangsu, China) and resuspended in 195 μL annexin-binding buffer. Then cells were incubated with 5 μL Annexin V and 10 μL PI in the dark for 15 min. Apoptotic cells were detected by BD LSRFortessa flow cytometer (BD Biosciences, Franklin Lakes, NJ, USA) and the apoptotic rates were calculated by FlowJo software (v10.0.7r2, BD Biosciences, Franklin Lakes, NJ, USA).

### 2.5. Determination of ER Stress, UPR Signaling Pathways and Apoptosis Related Proteins by Western Blot

The preparatory work for western blot including: extracting total proteins by the histiocyte lysate (RIPA: PMSF = 9:1, *v*/*v*, Beyotime Biotechnology, Jiangsu, China) for 20 min in an ice bath after cell treatments, determining the concentration of total proteins by BCA Protein Assay Kit (Beyotime Biotechnology, Jiangsu, China), and boiling the proteins for 5 min with SDS-PAGE Sample Loading Buffer (Beyotime Biotechnology, Jiangsu, China). All protein samples were stored at −80 °C for usage.

For western blot, each sample with 30 μg proteins was separated in SDS PAGE gel (6% or 10%, Beyotime Biotechnology, Jiangsu, China), running the gel at 80 V for 30 min and switching to 100 V for 60 min, and then the proteins were transferred to Polyvinylidene Fluoride (PVDF) membranes (0.22 μm, Millipore Corporation, Billerica, MA, USA) by electro-transfer at 100 V for 100 min. The blotting membranes were blocked with 5% (m/m) non-fat milk (Beyotime Biotechnology, Jiangsu, China) for 120 min at room temperature and then were incubated with the primary antibodies in an appropriate dilution (GRP78 (1:1000), ATF4 (1:1000), ATF6 (1:1000), phosphorylated-eIF2α (1:1000), CHOP (1:1000), Bim (1:1000), Bax (1:1000)) at 4 °C overnight and then incubated with the HRP-conjugated secondary antibody (1:2000) for 1 h at room temperature. All of the antibodies were purchased from Cell Signaling Technology (CST, Danvers, MA, USA). Then the blots were visualized with BeyoECL Plus ^TM^ reagent (Beyotime Biotechnology, Jiangsu, China) and detected by ECL detection system. The protein levels were analyzed by ImageJ software (1.52v, National Institutes of Health, Bethesda, MD, USA). We used ImageJ software to subtract the background of the western blot image and calculate the mean density of each protein sample. Then the density of the target protein was standardized by divided by the mean density of β actin. Subsequently, the density of each group was compared with the control group to obtain the relative change level, which was convenient for the comparison between different repeated experiments. The relative change level were the quantitative data for further analysis.

### 2.6. Determination of GRP78 by Immunofluorescence Staining

After the treatments of FLC, cells were fixed with 4% paraformaldehyde (PFA, Boster Biological Technology, Wuhan, China) for 10 min at room temperature and incubated with the primary antibody (GRP78, 1:200, CST) and Alexa Flour 488-labled secondary antibody (1:1000, CST). Cell nuclei were stained with DAPI Staining Solution (Beyotime Biotechnology, Jiangsu, China). Images were obtained using a fluorescence microscope and counted in five different fields each group with a 400× magnification. The fluorescence intensity was analyzed by ImageJ software.

### 2.7. Statistical Analysis

There were 6 replicates in Western blot and 3 replicates in flow cytometry experiment. All data was described as mean ± SD and analyzed with the GraphPad Prism 8.3.0 software (GraphPad Software, Inc., San Diego, CA, USA). The difference of treatment groups with the corresponding controls was analyzed using One-way analysis of variance (ANOVA) followed by LSD *t*-test. * *p* < 0.05, ** *p* < 0.01, *** *p* < 0.001, **** *p* < 0.0001.

## 3. Results

### 3.1. FLC Inhibited Cell Viability and Induced Cytotoxicity in TM4 Cells

**Figure 1:**
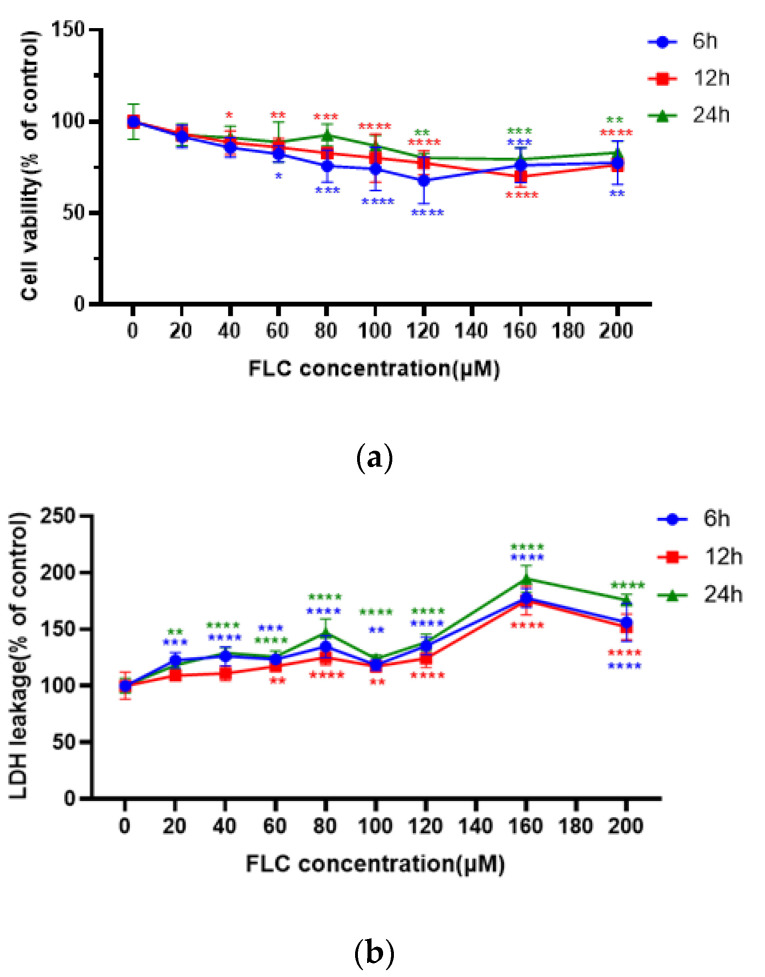
FLC inhibited cell viability and induced cytotoxicity in TM4 cells. TM4 cells were treated with serial concentrations of FLC (20–200 μM) for 6, 12, 24 h. (**a**) Cell viability (6, 12, 24 h) was detected by CCK-8 assay; (**b**) Cytotoxicity (6, 12, 24 h) was detected by LDH assay. * *p* < 0.05, ** *p* < 0.01, *** *p* < 0.001, **** *p* < 0.0001 vs. control group (0 μM FLC), *n* = 6.

After treated with FLC at different concentrations from 20 to 200 μM for 6, 12, 24 h, cell viability was detected by CCK-8 assay and cytotoxicity was detected by LDH assay. The results showed that the cell viability of TM4 cells was significantly inhibited in dose-dependent, and began to decline at 40 μM, 12^th^ hour. Cell viability was more damaged at 6^th^ hour than 12^th^ and 24^th^ hour in time relationship. (Figure 1a). The cytotoxicity of TM4 cells was also significantly increased dose-dependent and increased at 20 μM, 6^th^ and 24^th^ hour and more seriously at 160 μM, 12^th^ hour. Cytotoxicity was more damaged at 12^th^ hour than 6^th^ and 24^th^ hour in time relationship. (Figure 1b).

### 3.2. FLC Induced Apoptosis and Up-Regulated the Level of Pro-Apoptotic Proteins

**Figure 2:**
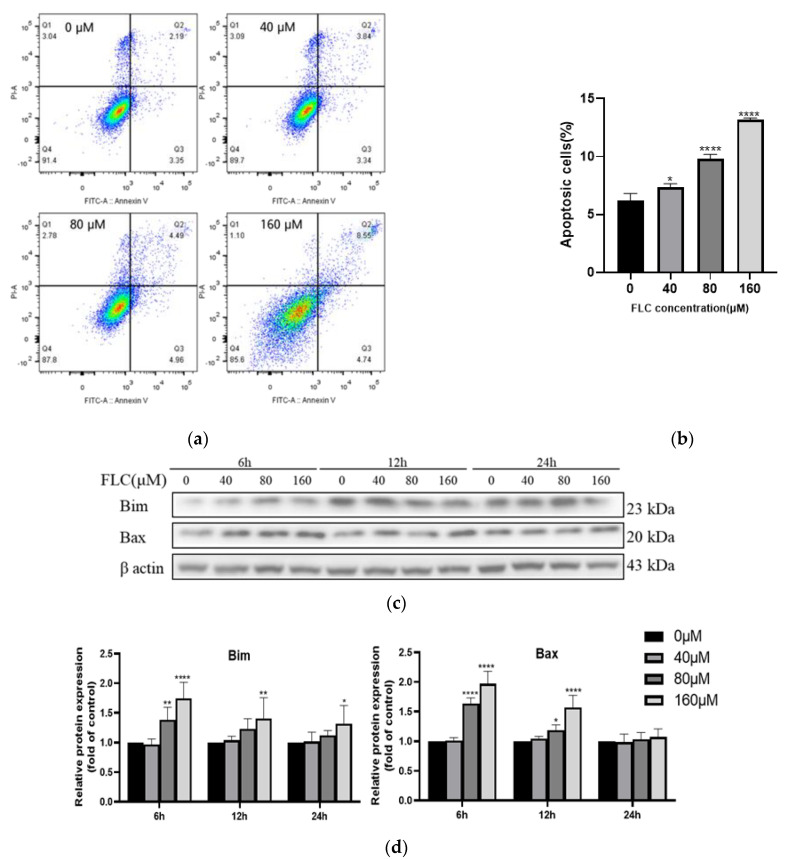
FLC induced apoptosis and altered up-regulated the level of pro-apoptotic proteins. TM4 cells were treated by FLC at the concentrations of 0, 40, 80, 160 μM for 6, 12, 24h. Apoptotic cells (6 h) were detected by flow cytometry and apoptosis protein levels (6, 12, 24 h) were determined by Western Blot with indicated antibodies. (**a**) The apoptotic cells were detected by Annexin V-PI double staining using Flow cytometer; (**b**) The quantitative data was analyzed by FlowJo and GraphPad Prism software (*n* = 3); (**c**) The representative blots were shown; (**d**) The quantitative data was analyzed by ImageJ and GraphPad Prism software (*n* = 6). * *p* < 0.05, ** *p* < 0.01,**** *p* < 0.0001 vs. control group (0 μM FLC).

To further prove the impair of FLC on TM4 cells, the apoptotic cells were detected by flow cytometry after treated with FLC at 0, 40, 80, 160 μM for 6 h. Positioning of quadrants on Annexin V/PI dot plots was performed (Figure 2a) and living cells (Annexin V^−^/PI^−^), early apoptotic cells (Annexin V^+^/PI^−^), late apoptotic cells (Annexin V^+^/PI^+^) and the necrotic cells (Annexin V^−^/PI^+^) were distinguished. The proportion of apoptotic cells was 6.2% ± 0.6%, 7.3% ± 0.3%, 9.8% ± 0.4%, 13.2% ± 0.2%, respectively, and increased in dose-dependent (Figure 2b). The levels of pro-apoptotic proteins (Bim and Bax) were up-regulated in dose-dependent (Figure 2c,d). The protein level of Bim was up-regulated in 6, 12, 24 h while Bax was up-regulated just in 6 and 12 h. There was no significant up-regulation in 24 h for Bax.

### 3.3. FLC Induced Endoplasmic Reticulum (ER) Stress and Activated Unfolded Protein Response (UPR) Signaling Pathways

**Figure 3:**
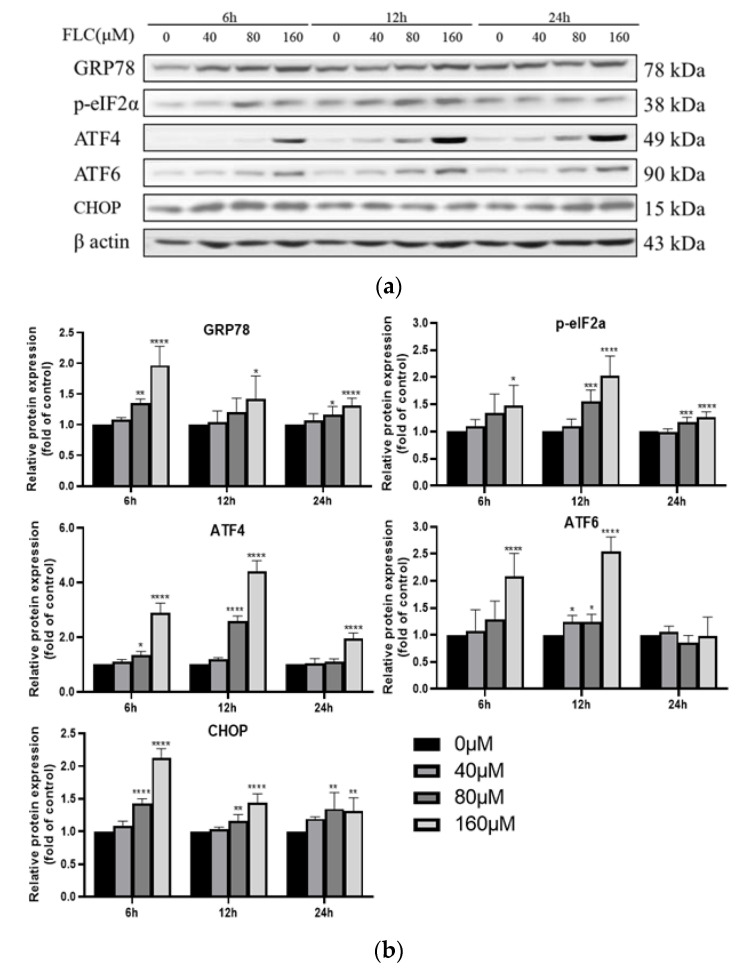

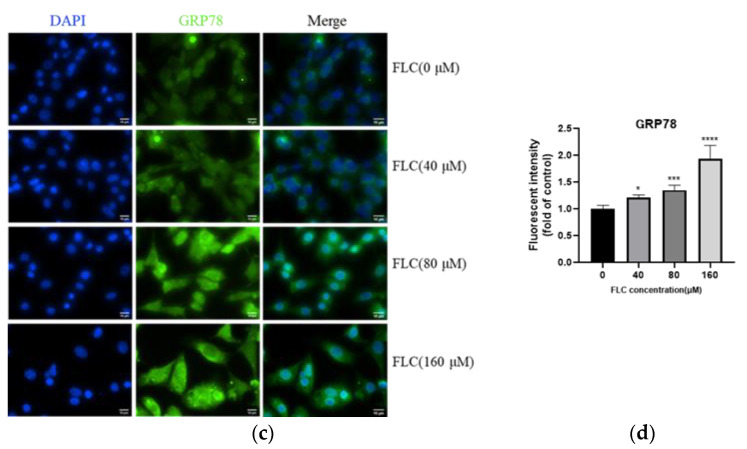
FLC induced Endoplasmic Reticulum (ER) stress and activated Unfolded Protein Response (UPR) signaling pathways. TM4 cells were treated by FLC at the concentrations of 0, 40, 80, 160 μM for 6, 12, 24 h. ER stress and UPR related protein levels were determined by Western Blot with indicated antibodies. (**a**) The representative blots were shown; (**b**) The quantitative data was analyzed by ImageJ and GraphPad Prism software. GRP78 protein level were detected by immunofluorescence staining in green and the nuclear was stained with DAPI in blue; (**c**) The representative images of GRP78 (Magnification, 400×; scale bar, 10 μm) were shown; (**d**) The quantitative data was analyzed by ImageJ and GraphPad Prism software. * *p* < 0.05, ** *p* < 0.01, *** *p* < 0.001, **** *p* < 0.0001 vs. control group (0 μM FLC), *n* = 6.

To explore the potential involvement of ER stress in FLC-induced TM4 cell apoptosis, the UPR signaling pathway proteins (GRP78, phosphorylated-eIF2α, ATF4, ATF6) were determined by Western Blot (Figure 3a). The expression of ER stress marker protein (GRP78) was up-regulated, and downstream proteins (phosphorylated-eIF2α, ATF4, ATF6) were activated over time (Figure 3b). To observe the up-regulated expression of GRP78, the protein was detected with specific antibody and observed by a fluorescence microscope, which was in green and the nuclear was in blue (400×) (Figure 3c). The fluorescence intensity was stronger significantly in FLC-treated groups (Figure 3d).

### 3.4. FLC Upregulated the Protein Level of CHOP

CHOP is a key molecular in ER stress-mediated apoptosis, and it is necessary to investigate whether CHOP is involved in FLC-induced apoptosis. The protein level of CHOP was also examined by Western Blot (Figure 3a) and it was up-regulated in dose- dependent (Figure 3b).

### 3.5. ISRIB Inhibited UPR Signaling Pathways and Alleviated ER Stress

**Figure 4:**
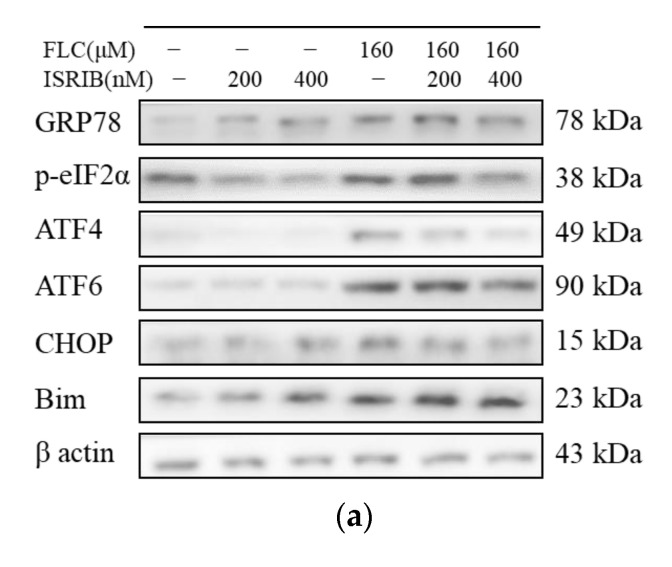

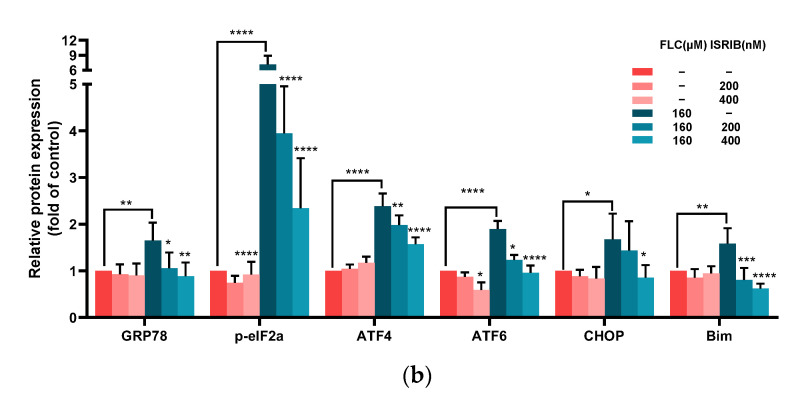
ISRIB inhibited UPR signaling pathways and ER stress. TM4 cells were treated by ISRIB at the concentration of 200, 400 nM for 6 h before treated by FLC at the concentration of 160 μM for 6 h. UPR and apoptosis related protein levels were determined by Western Blot with indicated antibodies. (**a**) The representative blots were shown; (**b**) The quantitative data was analyzed by ImageJ and GraphPad Prism software. * *p* < 0.05, ** *p* < 0.01, *** *p* < 0.001, **** *p* < 0.0001 vs. corresponding group, *n* = 6.

To determine the role of UPR signaling pathways played in FLC-induced TM4 cell apoptosis, we used 200 and 400 nM ISRIB (the phosphorylation inhibitor of eIF2α) to pre-treat TM4 cells for 6 h. After then, TM4 cells were treated with FLC for another 6 h, and the expression of ER stress and UPR signaling pathways related proteins (GRP78, phosphorylated-eIF2α, ATF4, ATF6), CHOP and the pro-apoptotic protein (Bim) were determined by Western Blot (Figure 4a). ISRIB inhibited the phosphorylation of eIF2α and the up-regulation of ER stress and apoptosis related proteins induced by FLC (Figure 4b).

### 3.6. ISRIB Alleviated FLC-Induced Cytotoxicity and Apoptosis

**Figure 5:**
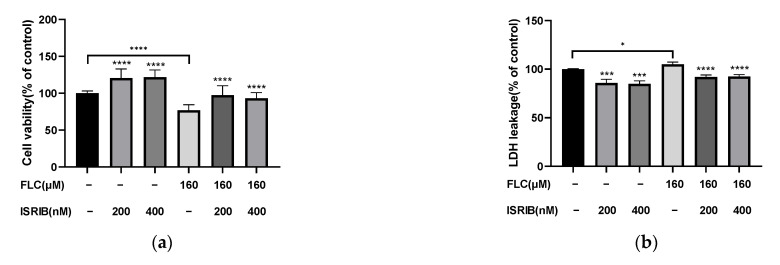

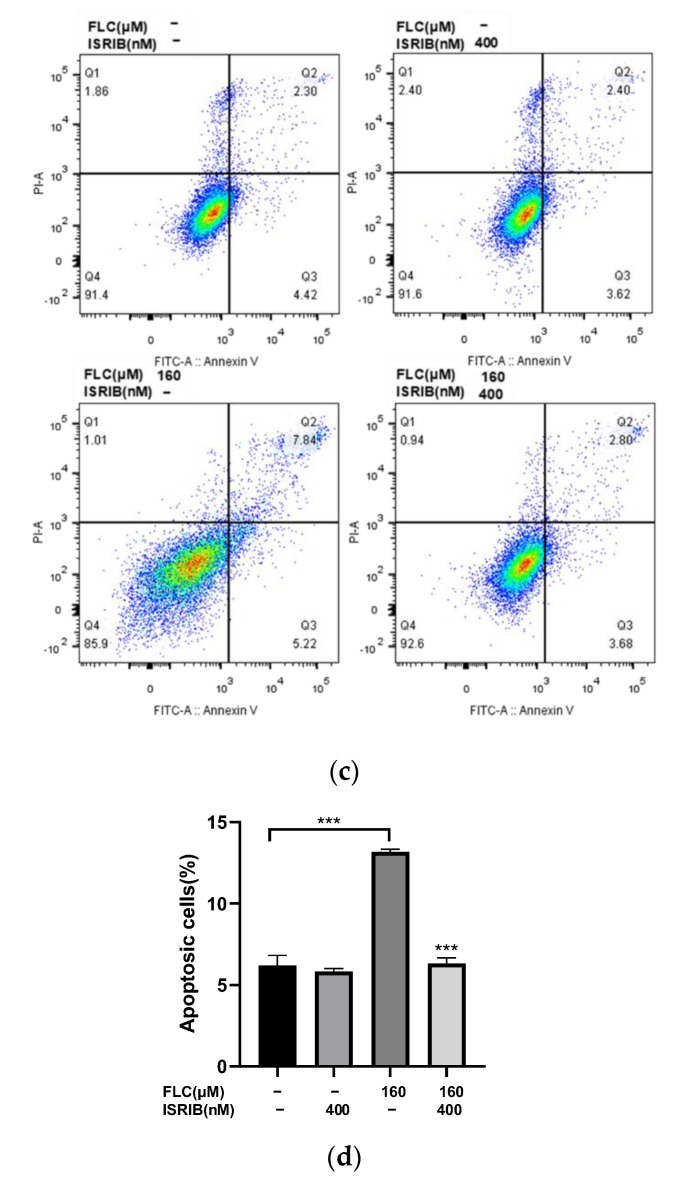
ISRIB alleviated FLC-induced cytotoxicity and apoptosis. TM4 cells were treated by ISRIB at the concentration of 200 and 400 nM for 6 h before treated by FLC at the concentration of 160 μM for 6 h. (**a**) Cell viability was detected by CCK-8 assay (*n* = 6); (**b**) Cytotoxicity was detected by LDH leakage assay (*n* = 6). Apoptotic cells were determined by flow cytometry; (**c**) The representative images were shown; (**d**) The quantitative data was analyzed by FlowJo and GraphPad Prism software (*n* = 3). * *p* < 0.05, *** *p* < 0.001, **** *p* < 0.0001 vs. corresponding group.

Then we detected the cytotoxicity and apoptosis induced by FLC once again to determine the impact of ISRIB in alleviating cell damage. The Cell viability was increased significantly (Figure 5a) and the cytotoxicity was alleviated significantly (Figure 5b). The percentage of apoptotic cells were decreased significantly (Figure 5c,d). ISRIB could alleviated the cytotoxicity induced by FLC in TM4 cells via inhibiting ER stress and UPR signaling pathways.

## 4. Discussion

The impair of herbicides on male reproduction and testicular toxicity has been concerned [30,31,32] and many studies focus on Sertoli cells since they are crucial important for homeostasis of testis by establishing blood-testis barrier and secreting active substance [10]. As a novel herbicide, FLC has been widely used in many countries and studies have shown that the target cell of FLC may be Sertoli cell for it can induce ROS cumulative damage [17,18,33], and disturb calcium homeostasis [16], which suggests the possible occurrence of ER stress [34]. In this study, the role of ER stress in FLC-induced apoptosis of mouse TM4 Sertoli cells was explored. It was found that FLC could activate ER stress and unfolded protein response (UPR), promote the phosphorylation of eIF2α, up-regulate the protein levels of ATF4 and ATF6, and promote the protein expression of CHOP, Bim and Bax, finally induce apoptosis. The inhibitor of eIF2α phosphorylation, ISRIB, can effectively inhibit UPR signaling pathway (eIF2α-ATF4-CHOP), alleviate the cytotoxicity and apoptosis induced by FLC, and protect TM4 cells from the toxic effects of FLC.

Cell viability began to decline at 40 μM FLC for 12 h, while cytotoxicity detected by LDH leakage assay began to increase at 20 μM for 6 h, suggesting that cytotoxicity can occur when TM4 cells were treated by lower dose of FLC for short time, which is consistent with the results of previous study in rat primary Sertoli cells [16]. The result of LDH leakage assay had a peak in 24 h since the content of LDH released by cells in the culture medium would accumulate along with prolonged time. Overall, the results suggested that the damage induced by FLC in TM4 cells is in dose-dependent. Taken into account cell viability and cytotoxicity, we selected 40, 80 and 160 μM as the treated concentrations of FLC for further study. The percentage of apoptotic cells and the expression of pro-apoptotic proteins (Bim and Bax) were also increased in dose-dependent way. The pro-apoptotic proteins (Bim and Bax) can be activated by CHOP [35,36]. We further detected the protein levels of CHOP and it was increased in dose-dependent as expected. The results suggested that the apoptosis induced by FLC was mediated by CHOP in TM4 cells.

CHOP is a key molecule in the process of ER stress-induced apoptosis [25,37]. The expression of CHOP is regulated by UPR signaling pathways [38]. Under normally physiological conditions, the UPR related proteins bind to the ER chaperone binding GRP78 and are in an inactive state [39]. Once ER stress occurs, the dissociated UPR related proteins which were separated from GRP78 activate downstream proteins by three different ways, namely inositol-requiring enzyme 1 (IRE1), protein kinase RNA-like ER kinase (PERK) and activating transcription factor 6 (ATF6) [40]. PERK and ATF6 signaling pathways are reported to increase the expression of CHOP and then induce apoptosis [22,24,41]. In this study, the up-regulated protein levels of CHOP indicated that the UPR signaling pathways may be activated. To explore whether UPR was activated, the protein levels related to UPR signaling pathways were detected. The protein levels of phosphorylated-eIF2α, ATF4 and ATF6 were increased, indicating the ATF4 and ATF6 pathways were activated. Meanwhile, the expression level of ER stress marker protein GRP78 also increased in a dose-dependent manner, suggesting that FLC activated ER stress.

To verify whether ER stress and UPR signaling pathways are involved in FLC-induced apoptosis in TM4 cells, the inhibitor of eIF2α phosphorylation (ISRIB) was used in intervention experiment. ISRIB is a drug-like compound which can alleviate ER stress derive from interference with UPR signaling pathways in mouse models [42]. We noted that different concentrations of ISRIB were used in different experiments [43,44], and therefore we chose two concentrations of 200, 400 nM to pre-treat TM4 cells for 6 h. The results showed that ISRIB could inhibit the up-regulated levels of phosphorylated-eIF2α and ATF4 induced by FLC. Meanwhile, the expression of ATF6 also can be affected since the regulatory networks of UPR signaling pathways are fully integrated and the activation of ATF6 are associated with the expression of ATF4 [45]. As the suppression of UPR pretreated with ISRIB, the protein level of CHOP has also been down-regulating and the same changes took place in the levels of pro-apoptotic protein (Bim) in dose-dependent. The results of intervention experiments also showed that ISRIB could alleviate FLC-induced apoptosis and increase cell viability in TM4 cells. These findings confirmed that ER stress and UPR signaling pathways were involved in FLC-induced apoptosis in TM4 cells.

## 5. Conclusions

FLC impaired cell viability and induced cytotoxicity and apoptosis mediated by ER stress via activating eIF2α-ATF4/ATF6-CHOP-Bim/Bax signaling pathways inTM4 cells. This study proved the possible mechanism of FLC-induced apoptosis in TM4 cells and provided a reliable direction for further study in vivo to verify ER stress maybe a target to alleviate the toxic effects of FLC.

## Data Availability

Not applicable.

## References

[1] Kaya A., Yigit E. (2014). The physiological and biochemical effects of salicylic acid on sunflowers (*Helianthus annuus*) exposed to flurochloridone. Ecotoxicol. Environ. Saf..

[2] EFSA (2010). Conclusion on the peer review of the pesticide risk assessment of the active substance Flurochloridone (notified active substance). EFSA J..

[3] Nikoloff N., Natale G.S., Marino D., Soloneski S., Larramendy M.L. (2014). Flurochloridone-based herbicides induced genotoxicity effects on Rhinella arenarum tadpoles (Anura: Bufonidae). Ecotoxicol. Environ. Saf..

[4] Nikoloff N., Soloneski S., Larramendy M.L. (2012). Genotoxic and cytotoxic evaluation of the herbicide flurochloridone on Chinese hamster ovary (CHO-K1) cells. Toxicol. In Vitro.

[5] Nikoloff N., Larramendy M.L., Soloneski S. (2014). Assessment of DNA damage, cytotoxicity, and apoptosis in human hepatoma (HepG2) cells after flurochloridone herbicide exposure. Food Chem. Toxicol..

[6] Zhang S., Cheng X., Wang Y., Fan J., Li R., Zhou S., Liu S., Shi J., Sun J., Hu Y. (2015). Ninety day toxicity and toxicokinetics of fluorochloridone after oral administration in rats. Int. J. Environ. Res. Public Health.

[7] Zhu H., Li R., Zhou S., Zhang S., Wang Y., Liu S., Song Q., Chang X., Zhang Y., Liu L. (2019). The Oral NOAEL of Flurochloridone in Male Wistar Rats in Ninety-Day Subchronic Toxicity Test Was 3 mg/kg/day. Int. J. Environ. Res. Public Health.

[8] Mruk D.D., Cheng C.Y. (2015). The Mammalian Blood-Testis Barrier: Its Biology and Regulation. Endocr. Rev..

[9] Larose H., Kent T., Ma Q., Shami A.N., Harerimana N., Li J.Z., Hammoud S.S., Handel M.A. (2020). Regulation of meiotic progression by Sertoli-cell androgen signaling. Mol. Biol. Cell.

[10] Wu S., Yan M., Ge R., Cheng C.Y. (2020). Crosstalk between Sertoli and Germ Cells in Male Fertility. Trends Mol. Med..

[11] Alves M.G., Neuhaus-Oliveira A., Moreira P.I., Socorro S., Oliveira P.F. (2013). Exposure to 2,4-dichlorophenoxyacetic acid alters glucose metabolism in immature rat Sertoli cells. Reprod. Toxicol..

[12] Gorga A., Rindone G.M., Centola C.L., Sobarzo C., Pellizzari E.H., Camberos M.D.C., Cigorraga S.B., Riera M.F., Galardo M.N., Meroni S.B. (2020). In vitro effects of glyphosate and Roundup on Sertoli cell physiology. Toxicol. In Vitro.

[13] Mao B., Mruk D., Lian Q., Ge R., Li C., Silvestrini B., Cheng C.Y. (2018). Mechanistic Insights into PFOS-Mediated Sertoli Cell Injury. Trends Mol. Med..

[14] Zhang X., Wang X., Liu T., Mo M., Ao L., Liu J., Cao J., Cui Z. (2018). ZnSO_4_ rescued vimentin from collapse in DBP-exposed Sertoli cells by attenuating ER stress and apoptosis. Toxicol. In Vitro.

[15] Zheng W., Wang B., Si M., Zou H., Song R., Gu J., Yuan Y., Liu X., Zhu G., Bai J. (2018). Zearalenone altered the cytoskeletal structure via ER stress- autophagy- oxidative stress pathway in mouse TM4 Sertoli cells. Sci. Rep..

[16] Liu L., Chang X., Zhang Y., Wu C., Li R., Tang L., Zhou Z. (2018). Fluorochloridone induces primary cultured Sertoli cells apoptosis: Involvement of ROS and intracellular calcium ions-mediated ERK1/2 activation. Toxicol. In Vitro.

[17] Sun W., Ni Z., Li R., Chang X., Li W., Yang M., Zhou Z. (2021). Flurochloridone induces Sertoli cell apoptosis through ROS-dependent mitochondrial pathway. Ecotoxicol. Environ. Saf..

[18] Ni Z., Sun W., Li R., Yang M., Zhang F., Chang X., Li W., Zhou Z. (2021). Fluorochloridone induces autophagy in TM4 Sertoli cells: Involvement of ROS-mediated AKT-mTOR signaling pathway. Reprod. Biol. Endocrinol..

[19] Lebeaupin C., Proics E., de Bieville C.H., Rousseau D., Bonnafous S., Patouraux S., Adam G., Lavallard V.J., Rovere C., Le Thuc O. (2015). ER stress induces NLRP3 inflammasome activation and hepatocyte death. Cell Death Dis..

[20] Coleman O.I., Haller D. (2019). ER Stress and the UPR in Shaping Intestinal Tissue Homeostasis and Immunity. Front. Immunol..

[21] Hetz C., Saxena S. (2017). ER stress and the unfolded protein response in neurodegeneration. Nat. Rev. Neurol..

[22] Hetz C., Zhang K., Kaufman R.J. (2020). Mechanisms, regulation and functions of the unfolded protein response. Nat. Rev. Mol. Cell Biol..

[23] Han X., Zhang P., Jiang R., Xia F., Li M., Guo F.J. (2020). Retraction Note to: Explore on the effect of ATF6 on cell growth and apoptosis in cartilage development. Histochem. Cell Biol..

[24] Zhang S., Zhao X., Hao J., Zhu Y., Wang Y., Wang L., Guo S., Yi H., Liu Y., Liu J. (2021). The role of ATF6 in Cr(VI)-induced apoptosis in DF-1 cells. J. Hazard Mater..

[25] Ge J., Sun H., Li J., Shan Y., Zhao Y., Liao F., Yang Y., Cui X., Liu Z. (2019). Involvement of CHOP in activin Ainduced myeloma NS1 cell apoptosis. Oncol. Rep..

[26] Iurlaro R., Munoz-Pinedo C. (2016). Cell death induced by endoplasmic reticulum stress. FEBS J..

[27] Puthalakath H., O’Reilly L.A., Gunn P., Lee L., Kelly P.N., Huntington N.D., Hughes P.D., Michalak E.M., McKimm-Breschkin J., Motoyama N. (2007). ER stress triggers apoptosis by activating BH3-only protein Bim. Cell.

[28] Vanlaeys A., Dubuisson F., Seralini G.E., Travert C. (2018). Formulants of glyphosate-based herbicides have more deleterious impact than glyphosate on TM4 Sertoli cells. Toxicol In Vitro.

[29] Li Z., Wang H., Huang S., Zhou L., Wang L., Du C., Wang C. (2015). Establishment of stable MRP1 knockdown by lentivirus-delivered shRNA in the mouse testis Sertoli TM4 cell line. Toxicol. Mech. Methods.

[30] Li H., Zhu Q., Wang S., Huang T., Li X., Ni C., Fang Y., Li L., Lian Q., Ge R.S. (2019). Paraquat exposure delays stem/progenitor Leydig cell regeneration in the adult rat testis. Chemosphere.

[31] Sai L., Qu B., Zhang J., Liu J., Jia Q., Bo C., Zhang Y., Yu G., Han R., Peng C. (2019). Analysis of long non-coding RNA involved in atrazine-induced testicular degeneration of Xenopus laevis. Environ. Toxicol..

[32] Krzastek S.C., Farhi J., Gray M., Smith R.P. (2020). Impact of environmental toxin exposure on male fertility potential. Transl. Androl. Urol..

[33] Zhou S., Li R., Hou W., Wang Y., Zhang S., Yu Y., Zhang L., Zhu H., Zhang Z., Fang J. (2020). RNA-seq analysis of testes from flurochloridone-treated rats. Toxicol. Mech. Methods.

[34] Martucciello S., Masullo M., Cerulli A., Piacente S. (2020). Natural Products Targeting ER Stress, and the Functional Link to Mitochondria. Int. J. Mol. Sci..

[35] Shigemi Z., Manabe K., Hara N., Baba Y., Hosokawa K., Kagawa H., Watanabe T., Fujimuro M. (2017). Methylseleninic acid and sodium selenite induce severe ER stress and subsequent apoptosis through UPR activation in PEL cells. Chem. Biol. Interact.

[36] Akazawa Y., Cazanave S., Mott J.L., Elmi N., Bronk S.F., Kohno S., Charlton M.R., Gores G.J. (2010). Palmitoleate attenuates palmitate-induced Bim and PUMA up-regulation and hepatocyte lipoapoptosis. J. Hepatol..

[37] Panganiban R.A., Park H.R., Sun M., Shumyatcher M., Himes B.E., Lu Q. (2019). Genome-wide CRISPR screen identifies suppressors of endoplasmic reticulum stress-induced apoptosis. Proc. Natl. Acad. Sci. USA.

[38] Mota M., Banini B.A., Cazanave S.C., Sanyal A.J. (2016). Molecular mechanisms of lipotoxicity and glucotoxicity in nonalcoholic fatty liver disease. Metabolism.

[39] Kopp M.C., Larburu N., Durairaj V., Adams C.J., Ali M. (2019). UPR proteins IRE1 and PERK switch BiP from chaperone to ER stress sensor. Nat. Struct. Mol. Biol..

[40] Moncan M., Mnich K., Blomme A., Almanza A., Samali A., Gorman A.M. (2021). Regulation of lipid metabolism by the unfolded protein response. J. Cell Mol. Med..

[41] Read A., Schroder M. (2021). The Unfolded Protein Response: An Overview. Biology (Basel).

[42] Zyryanova A.F., Kashiwagi K., Rato C., Harding H.P., Crespillo-Casado A., Perera L.A., Sakamoto A., Nishimoto M., Yonemochi M., Shirouzu M. (2021). ISRIB Blunts the Integrated Stress Response by Allosterically Antagonising the Inhibitory Effect of Phosphorylated eIF2 on eIF2B. Mol. Cell.

[43] Rabouw H.H., Langereis M.A., Anand A.A., Visser L.J., de Groot R.J., Walter P., van Kuppeveld F. (2019). Small molecule ISRIB suppresses the integrated stress response within a defined window of activation. Proc. Natl. Acad. Sci. USA.

[44] Bugallo R., Marlin E., Baltanas A., Toledo E., Ferrero R., Vinueza-Gavilanes R., Larrea L., Arrasate M., Aragon T. (2020). Fine tuning of the unfolded protein response by ISRIB improves neuronal survival in a model of amyotrophic lateral sclerosis. Cell Death Dis..

[45] Teske B.F., Wek S.A., Bunpo P., Cundiff J.K., McClintick J.N., Anthony T.G., Wek R.C. (2011). The eIF2 kinase PERK and the integrated stress response facilitate activation of ATF6 during endoplasmic reticulum stress. Mol. Biol. Cell.

